# Feasibility of Real Time Internet-Based Teleconsultation in Patients With Multiple Sclerosis: Interventional Pilot Study

**DOI:** 10.2196/18178

**Published:** 2020-08-13

**Authors:** Miguel D'Haeseleer, Piet Eelen, Nima Sadeghi, Marie B D'Hooghe, Jeroen Van Schependom, Guy Nagels

**Affiliations:** 1 Universitair Ziekenhuis Brussel Brussels Belgium; 2 Nationaal Multiple Sclerose Centrum Melsbroek Belgium; 3 Center for Neurosciences Vrije Universiteit Brussel Brussels Belgium; 4 Zebra Academy Brussels Belgium

**Keywords:** multiple sclerosis, teleconsultation, internet, feasibility, eHealth

## Abstract

**Background:**

Telemedicine (TM) is currently flourishing in rural and emergency settings, but its implementation in the routine management of chronic neurological disorders has developed with more hesitation. Limited access to specialized care facilities and expanding patient populations, combined with unprecedented mobility restrictions imposed by the coronavirus disease pandemic, are currently stressing the need for remote solutions in this field. Studies in patients with multiple sclerosis (MS) have been heterogeneous in objectives and methodology but generally support the concept that TM interventions produce clinical benefits, cost-effectiveness, and user satisfaction. Nonetheless, data on live interaction between patients and health care providers for MS teleconsultation purposes remain scarce.

**Objective:**

The aim of this study is to demonstrate the feasibility of planned real time audiovisual teleconsultation over the internet for patients with MS.

**Methods:**

A total of 20 patients with MS presenting at a specialized MS center in Belgium were recruited for this study. One teleconsultation was scheduled for each participant. Patients were provided a unique hyperlink by mail in advance, leading them automatically and directly to the virtual waiting room, where they could accept or decline our incoming call. All teleconsultations were performed by a trained medical student with the intention to keep the conversation similar to what is usually discussed during a classic face-to-face MS consultation; no remote physical exams were performed. The approach was considered feasible if at least 80% of the planned TM visits could be successfully completed at the foreseen moment. Patient satisfaction (technical quality, convenience, and overall quality of care) was evaluated at the end of each teleconsultation by means of 5-point Likert scales containing the categories very unsatisfied, unsatisfied, neutral, satisfied, and highly satisfied.

**Results:**

Out of 20 consultations, 17 were successfully completed (85%). Failures were due to patients not responding (n=2) and technical issues (n=1). Out of the 17 consultations, 17 patients declared themselves satisfied or highly satisfied for technical quality, 15 patients for convenience, and 16 patients for overall quality of care.

**Conclusions:**

Planned real time audiovisual teleconsultation over the internet is feasible and highly appreciated in patients with MS. Incorporation of such services in routine clinical MS practice is expected to improve access to specialized care facilities for affected patients.

## Introduction

Telemedicine (TM) is defined as the exchange of medical information between patients and health care providers in distinct locations using electronic communication technology [1]. Its primary aim is to improve public health by allowing medical services that cannot be easily established in a face-to-face manner. TM can, from a practical perspective, essentially be broken down into three major categories: real time interactive sessions (eg, telephone conversations, videoconferences), store-and-forward technology (eg, email), and remote monitoring (eg, self-assessment devices) [2]. Most efforts so far have been devoted to the implementation of TM in rural and emergency medicine, as illustrated by the successful example of telestroke [3], while common chronic neurological disorders seem to have adopted digital health technology with more hesitation.

Nervous system diseases are the leading cause of disability around the world, and their burden is expected to double over the next 25 years, an evolution mainly driven by expansion of the aging population [4]. Current access to neurological facilities is limited already, and classic care models will likely not be able to match a future demand of that magnitude [5]. Several high-impact papers have recently highlighted the potential of TM to help close this gap and to prove the benefits for millions of people with chronic conditions such as epilepsy, headache syndromes, dementia, movement disorders, and multiple sclerosis (MS) [5-7]. Accelerated and wide-scale incorporation of digital health services is generally, and more specifically in the field of neurology, expected and even recommended, viewpoints that have recently been sharpened by the coronavirus disease [8] outbreak, where social isolation has been deployed as the primary measure of constraint [9-12].

Individuals with MS typically receive their diagnosis during young adulthood [13], when busy social and professional schedules risk to limit the time available for medical attention. Strikingly, a high percentage of them appear to be interested in online interaction with health care providers and peers [14], consistent with the infodemiological finding (infodemiology refers to a newly described area of epidemiological research that deals with the question of how health information is accessed on the internet [15]) that an ever-increasing part of the general population exploits the internet to obtain health information [16]. Studies in patients with MS have applied different types of TM and were conducted for various purposes (eg, management, disability assessment, treatment, rehabilitation). A comprehensive and structured review of the literature has recently been published by Yeroushalmi and colleagues [17], in which the authors conclude that most randomized controlled trials have demonstrated either no difference between a TM versus an in-person intervention or an association of the former with a more beneficial outcome. Furthermore, the majority of these studies were considered low to medium cost. Another recent overview has primarily focused on the potential of digital communication tools to integrate real-world patient-centered data into clinical MS registries, which could then be easily shared between caregivers and lead, in conjunction with machine learning and artificial intelligence, to improved disease understanding, decision support, and self-empowerment [18]. Nonetheless, data on live interaction between patients and health care providers in a teleconsultation setting are relatively scarce. The main objective of our study is to explore whether planned real time audiovisual teleconsultation over the internet is feasible in patients with MS.

## Methods

### Ethics

This study was approved by the ethics committee of the Nationaal MS Center in Melsbroek (local; internal reference: AvN/AVDZ) and the Universitair Ziekenhuis Brussel (leading; internal reference: 2018/269 - Belgian Unique Number: 143201836797). Written informed consent was obtained from all participants prior to inclusion.

### Patient Cohort

A total of 20 French- or Dutch-speaking patients with MS, according to the 2017 revised McDonald criteria [19], were recruited at the Nationaal MS Center in Melsbroek. Home access to the internet with a webcam-equipped device was required for inclusion. Recruitment of individuals with a high suspicion of moderate to severe cognitive impairment (based on common sense judgement of the medical record or initial patient contact) was actively avoided. A known history of cognitive dysfunction (defined as scoring less than 21 on the Mini Mental State Evaluation, if present in the medical record; no cognitive evaluations were performed within this study) accounted as a formal exclusion criterion. Age, sex, clinical subtype (ie, relapsing-remitting versus secondary or primary progressive MS), and disability (the latter represented by Expanded Disability Status Scale [EDSS] scores, with higher scores representing a more pronounced degree of functional impairment [20]) were extracted as demographic variables from the local existing patient records. EDSS scores are routinely assessed at nearly every visit during regular neurological follow-ups in the Melsbroek center, and the most recent available result was consistently selected in each participant. The same accounted for the determination of the clinical MS subtype.

### Teleconsultations

**Figure 1:**
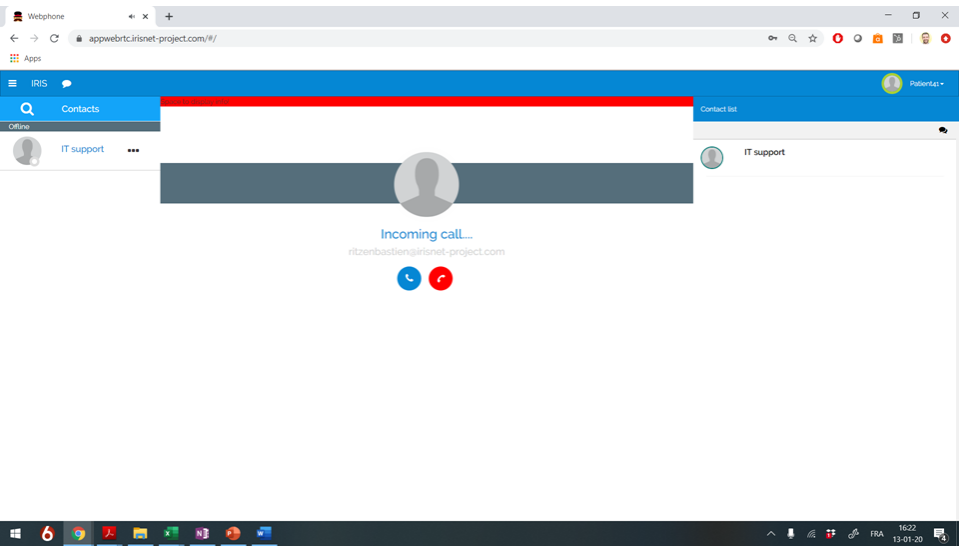
Zebra Academy teleconsultation platform. The screenshot is from the perspective of a fictional patient receiving our incoming internet call. Clicking on the blue icon will establish the connection. Bastien Ritzen is a member of the Zebra Academy crew (for illustrative purposes). IT: information technology.

One teleconsultation was scheduled for every participant, in addition to standard neurological follow-ups, and performed by a trained medical student (NS) under the supervision of the principle investigator of the study (MD), using a novel internet-based communication platform obtained from Zebra Academy. This technology was originally developed at the Vrije Universiteit Brussel with the purpose of supporting prehospital management of acute stroke [21]. A checklist (with questions about items such as general and neurological health, medication, and lifestyle factors; see Textbox 1) similar to the usual content of a classic face-to-face MS consultation was used as a backbone to guide the conversation, but deviations at the initiative of the patient were allowed. Patients were provided a unique hyperlink by mail in advance, leading them directly to the virtual waiting room where they could notice and accept our incoming call at the time of the scheduled appointment (Figure 1). Access was possible from any device with a webcam (ie, laptop, desktop, tablet, smartphone). Google Chrome was used as a web browser on both sides of the connection, as advised by Zebra Academy. All patients had to respond to the call within the next 30 minutes following the scheduled time, with a maximum of three attempts during this window. A report was written and forwarded to the treating neurologist after each visit to allow additional medical interventions outside of the scope and protocol of this study, if deemed necessary based on the content of the teleconsultation. The act of teleconsultation was considered feasible if at least 80% of the planned visits could be successfully completed.

**Checklist used to guide the teleconsultation.**

**Checklist**

- How would you describe your current general health status?
- Did you experience any relapses or neurological deterioration over the past 3 months?
- Did you experience any other medical problems (not related to multiple sclerosis [MS]) over this time period?
- Do you have any of the following MS-related symptoms: muscle weakness, sensory loss, visual disturbances, swallowing or speech difficulties, bladder or bowl dysfunction, pain or spasticity (muscle spasms), fatigue, mood swings, mental slowness?
- Which of these symptoms have recently progressed or currently have a significant impact on your quality of life?
- What medication do you take?
- Do you experience any side effects or other inconvenience related to your medical treatment?
- Do you follow a regular rehabilitation scheme? If so, please estimate the average time of weekly physical activity.
- Would you describe yourself as compliant to your medical and rehabilitation treatment? If not, please explain why.
- Do you smoke or use recreational drugs?
- How would you evaluate your professional performance? How many days of work did you miss over the past 3 months?
- How would you evaluate your social and family member interactions?

### Satisfaction

Patient satisfaction was evaluated at the end of each teleconsultation by means of 5-point Likert scales containing the categories very unsatisfied, unsatisfied, neutral, satisfied, and highly satisfied. Assessments were independently carried out for technical quality, convenience, and overall quality of care (QoC).

### Data Availability

Anonymized data will be shared by request from any qualified investigator.

## Results

### Patient Cohort

The median age and EDSS scores of the participants (as extracted from the Melsbroek patient database) at the time of inclusion were 41 (range 27-62) years of age and 4.0 (range 0.0-6.5), respectively. The female to male ratio was 11:9. A total of 14 participants had relapsing-remitting MS, while the other 6 had a progressive disease course (4/20 secondary and 2/20 primary progressive MS).

### Teleconsultations

A total of 17 (85%) out of 20 planned teleconsultations were successfully completed. Failures were due to participants not responding (n=2) and technical issues (n=1). The nonresponders were contacted at a later time by telephone and both let us know that they had forgotten the appointment. The technical issue was a blank video screen from the patient’s perspective, appearing after responding to each of the three allowed attempts to connect.

### Satisfaction

Out of the total 17 consultations, 17 patients declared themselves to be satisfied or highly satisfied with the teleconsultation for technical quality, 15 patients for convenience, and 16 patients for overall QoC.

## Discussion

We present the first study demonstrating feasibility of planned real time audiovisual teleconsultation over the internet in patients with MS, in which feasibility was a priori defined as the ability to complete a fixed number of scheduled TM visits at the foreseen moment. In addition, patient appreciation regarding the technical aspects and general approach was excellent. Our results are in line with a recent comparative crossover trial from Robb and colleagues [22], in which there was less than 15% difference between successfully performed internet-based video house calls and personal in-hospital visits (prespecified study target) after each participant agreed to receive both consultation modalities consecutively. Eventually 25 (67.6%) out of the 37 scheduled TM visits could be completed according to that protocol. Reasons for failure were not mentioned. The vast majority of participants reported that they would recommend teleconsultation to others (97.1%) and stated that establishing the virtual connection was easy (94.3%) [22]. To the best of our knowledge, there are no other studies exploiting this particular type of TM intervention. Previous efforts at connecting patients with health care providers, in the context of general MS management, were based on online texting, telephone hotlines, or noninternet videoconferencing outside the clinic hours [23-29]. Respective methodologies and objectives were heterogeneous but most of these studies had in common that telehealth services provided patient well-being and user satisfaction [17].

Our work was, to a certain extent, inspired by a previous randomized controlled trial demonstrating the feasibility of clinical monitoring over a 12-month period via video house calls in patients with Parkinson disease living throughout the United States. QoC perception was not significantly influenced, but the intervention did lead to a high degree of patient satisfaction and time gain [30]. Several reasons can be given to assume that patients with MS are at least as equally good candidates to benefit from such an approach. As already mentioned in the introduction, these individuals seem to have a strong interest in online communication, and the disease typically affects young adults who might have other priorities than seeking pathology-specific medical attention. In addition, MS commonly leads to cumulative and substantial physical disability [13], which can create additional logistic boundaries even in areas highly saturated with neurologists. Periodic assessment is crucial to monitor disease activity, treatment response, and health-related quality of life, and inevitably requires frequent visits to the neurology office. One study reported that nearly 30% of patients with MS do not receive neurological care at all, which undeniably decreases the likelihood of access to appropriate disease-modifying treatment and specialized facilities [31]. Over the past decades, it has become increasingly clear that absence or delayed start of an immunological maintenance therapy is a risk factor for a worse prognosis [32]. Interestingly, digital health services seem to have been positively welcomed by MS neurologists as well [33], for whom they can serve as a medium for education and case discussion [34,35].

Demonstrating feasibility is an early but essential hurdle toward the implementation of technological development in medicine. Various TM interventions have already been deployed to support medical management, disability assessment, treatment, and physical or cognitive rehabilitation in individuals with MS [17]. In contrast, real time audiovisual communication over the internet between health care providers and patients, aimed at routine clinical follow-up, is novel in this field. One of the most attractive features of the system applied in our study is its user-friendliness by simplicity, as it takes only two clicks from patients to participate in the call. On the other hand, we have to acknowledge that drawing conclusions from this pilot is not free from potential pitfalls. First, the total number of participants and scheduled TM visits was small. One should be particularly careful extrapolating our findings to the full community of individuals with MS, as results might be less positive when considering populations with more severe clinical disability. Previous work has revealed that patients with cognitive or visual impairment experienced more difficulties while using home-based TM systems [23,25], whereas we have actively avoided recruitment of patients with apparent cognitive dysfunction. Second, teleconsultations were performed by a medical student and did not include a physical exam. Uncertainty about the ability to conduct an accurate and detailed remote clinical neurological evaluation remains a general weakness of TM. However, it is worth mentioning that Bove and colleagues [36] recently reported agreement within 1 point between in-person and televideo-enabled EDSS scores for 88% of the cases, which is on par with the in-person interrater variability described by others. Third, feasibility was considered to be a single binary outcome measure (“either the teleconsultation could be successfully completed or not”), while other potentially interesting variables such as time- and cost-effectiveness have not been taken into account. Satisfaction was assessed for exploratory reasons with Likert scales, which are easy to use but lack validation for these particular trials. There are recent data suggesting that such scales may be more vulnerable to bias from confounding factors and that a ceiling effect may be more difficult to avoid when compared with a visual analogue scale [37]. Fourth, our study had a cross-sectional design and was not able to inform us on patient compliance or technical reliability over time. Longitudinal data regarding real time interactive patient monitoring are scarce, but an Israeli study suggested that regular videoconferencing over the telephone line over 6 months improves clinical outcomes while reducing medical expenses [29]. In a future research project, we will investigate the feasibility and potential of patient follow-ups with the Zebra communication platform over 1 year. Positive results may open new ways to explore TM technology for multiple purposes in MS (eg, validation of clinical rating scales, noninferiority trial designs in relapse evaluation and routine follow-up, remote virtual participation of neurologist in multidisciplinary consultation, and therapeutic group sessions such as mindfulness). It would also be of interest to explore the utility of TM for diagnostic counselling and to do comparative studies across distinct MS populations, as there might be variability in results due to geographical, cultural, or social differences. However, even with today’s knowledge, and if legal and reimbursement policies allow, incorporating TM in routine MS practice is likely to reduce barriers between affected patients and specialized care facilities, potentially leading to improved clinical prognosis and health-related quality of life in a cost- and time-efficient manner.

## Abbreviations

EDSS: Expanded Disability Status Scale
MS: multiple sclerosis
QoC: quality of care
TM: telemedicine
